# Supplementary material for: Types and clinical outcomes of chemical ingestion in emergency departments in South Korea (2011-2016)
Source: PLoS One. 2020 Mar 4;15(3):e0229939. doi: 10.1371/journal.pone.0229939 (PMC7055891; doi:10.1371/journal.pone.0229939)
Supplement: S1 Table — (DOCX) [file pone.0229939.s001.docx]

**S1 Table. Total incidence, admission and mortality of chemical ingestion in adult (>20 years) at ED, South Korea, 2011-2016**

|  | Total | Ad | Mo |  |  | Total | Ad | Mo |
| --- | --- | --- | --- | --- | --- | --- | --- | --- |
| Acetic acid | 16 | 5 | 0 |  | Light oil | 7 | 1 | 0 |
| Acetone | 16 | 1 | 0 |  | Lithium | 2 | 0 | 0 |
| Acrylic resin | 4 | 0 | 0 |  | Mercury[Hydrargyrum] | 6 | 1 | 0 |
| Alkylammoniumhydroxyd | 1 | 0 | 0 |  | Methanol | 44 | 21 | 1 |
| Alkyltrimethylammonium Chloride | 1 | 0 | 0 |  | Methylene chloride | 2 | 0 | 0 |
| Aluminium | 3 | 0 | 0 |  | Methylparaben | 1 | 0 | 0 |
| Ammonia | 5 | 3 | 0 |  | Mineral oil | 7 | 3 | 0 |
| Ammonium | 1 | 0 | 0 |  | Naphthalene | 4 | 1 | 0 |
| Ammonium Chloride | 2 | 1 | 1 |  | Nicotine | 10 | 3 | 0 |
| Ammonium phosphate | 8 | 1 | 0 |  | Nitrogen | 8 | 1 | 0 |
| Arsenic | 1 | 0 | 0 |  | Nitric acid | 13 | 2 | 0 |
| Bentonite | 2 | 1 | 0 |  | Other heavy metal | 3 | 1 | 0 |
| Benzene | 6 | 1 | 0 |  | Other hydrocarbon | 3 | 1 | 0 |
| Benzethonium chloride | 1 | 0 | 0 |  | Palladium | 1 | 0 | 0 |
| Benzol | 1 | 0 | 0 |  | Paraffin | 6 | 2 | 1 |
| Boric acid | 6 | 3 | 0 |  | Petroleum | 5 | 1 | 0 |
| Butane | 2 | 0 | 0 |  | Phenol | 11 | 1 | 0 |
| Butanol | 2 | 0 | 0 |  | Phenoxyethanol | 3 | 0 | 0 |
| Cadmium | 1 | 0 | 0 |  | Poly Ferric Sulfate Plus | 1 | 0 | 0 |
| Calcium carbonate | 4 | 1 | 0 |  | Polyethylene | 1 | 1 | 0 |
| Calcium chloride | 11 | 1 | 0 |  | Polyphenol | 2 | 1 | 0 |
| Calcium phosphate | 4 | 2 | 0 |  | Potassium Cyanide | 29 | 21 | 3 |
| Carbon tetrachloride | 1 | 0 | 0 |  | Potassium Chloride | 2 | 1 | 0 |
| Casein | 1 | 1 | 0 |  | Potassium Hydroxide | 2 | 0 | 0 |
| Chlorhexidine Digluconate | 1 | 1 | 0 |  | Potassium permanganate | 1 | 1 | 0 |
| Chlorine dioxide | 1 | 0 | 0 |  | Propylene Glycol | 1 | 0 | 0 |
| Chloroform | 3 | 0 | 0 |  | Silica | 3 | 1 | 0 |
| Cresol | 2 | 1 | 1 |  | Silicagel | 7 | 0 | 0 |
| Chromic acid | 1 | 1 | 0 |  | Silicic acid ester | 1 | 1 | 0 |
| Citric acid | 5 | 2 | 0 |  | Silicon | 3 | 1 | 0 |
| Copper sulfate | 3 | 2 | 1 |  | Sodium chlorite | 5 | 3 | 0 |
| Cyanide | 1 | 0 | 0 |  | Sodium fluoride | 4 | 0 | 0 |
| Cypermethrin | 1 | 0 | 0 |  | Sodium hydroxide | 139 | 49 | 3 |
| Dichloromethane | 1 | 1 | 0 |  | Sodium hydrogen carbonate | 5 | 2 | 0 |
| Dimethyl Ammonium | 1 | 0 | 0 |  | Sodium Hypochlorite | 919 | 313 | 10 |
| Epoxy | 10 | 4 | 0 |  | Sodium Lauroyl Sarcosinate | 3 | 0 | 0 |
| Ethanol | 195 | 31 | 1 |  | Sodium Lauryl Sulfate | 6 | 0 | 0 |
| Ethyl cyanoacrylate | 16 | 4 | 0 |  | Sodium Nitrite | 2 | 1 | 0 |
| Ethyl Lactate | 2 | 0 | 0 |  | Sodium Metaphosphate | 8 | 3 | 0 |
| Ethylene dichloride | 1 | 0 | 0 |  | Sodium nitrate | 1 | 0 | 0 |
| Ethylene glycol | 72 | 56 | 1 |  | Sodium peroxocarbonate | 16 | 3 | 0 |
| Fluorine | 2 | 0 | 0 |  | Sodium silicate | 12 | 3 | 0 |
| Formaldehyde | 11 | 1 | 0 |  | Solvent | 6 | 1 | 1 |
| Gasoline | 37 | 14 | 2 |  | Stearic Acid | 2 | 1 | 0 |
| Glacial acetic acid | 186 | 139 | 36 |  | Succinic acid | 1 | 0 | 0 |
| Glutaral | 1 | 1 | 0 |  | Surfactant | 313 | 71 | 7 |
| Glyphosate | 2 | 1 | 0 |  | Sulfur | 1 | 0 | 0 |
| Glyphosate-ammonium | 1 | 1 | 0 |  | Sulfuric acid | 38 | 5 | 1 |
| Glycerin | 2 | 0 | 0 |  | Texanol | 1 | 0 | 0 |
| Gold cyanide | 2 | 2 | 0 |  | Thionyl chloride | 1 | 0 | 0 |
| Gum Arabic | 3 | 1 | 0 |  | Thymol | 1 | 0 | 0 |
| Hydrochloric acid | 71 | 41 | 7 |  | Toluene | 133 | 28 | 2 |
| Hypochlorous acid | 4 | 1 | 0 |  | Toluene Diisocyanate | 1 | 0 | 0 |
| Hydrofluoric Acid | 66 | 3 | 0 |  | Trichloroacetic acid | 2 | 1 | 0 |
| Hydrogen chloride | 1 | 1 | 0 |  | Triethylamine | 1 | 0 | 0 |
| Hydrogen cyanide | 4 | 0 | 0 |  | Trifluoroacetyl acid | 1 | 0 | 0 |
| Hydrogen peroxide | 43 | 7 | 0 |  | Turpentine | 1 | 0 | 0 |
| Hydrogen sulfide | 7 | 6 | 0 |  | Urea | 2 | 1 | 0 |
| Imazaquin | 1 | 0 | 0 |  | Urethane | 7 | 2 | 0 |
| Iodine | 2 | 2 | 0 |  | Varnish | 1 | 0 | 0 |
| Iron oxide | 1 | 0 | 0 |  | Xylene | 3 | 0 | 0 |
| Isopropyl alcohol | 5 | 0 | 0 |  | 1-octanol | 1 | 0 | 0 |
| Kerosene | 16 | 4 | 0 |  | 1,2,3,4_Tetrahydroisoquinoline | 1 | 0 | 0 |
| Lead[Plumbum] | 1 | 0 | 0 |  |  |  |  |  |
|  |  |  |  |  | Total | 2712 | 903 | 79 |
|  |  |  |  |  | % | 100.0 | 33.3 | 2.9 |
