# Supplementary material for: Types and clinical outcomes of chemical ingestion in emergency departments in South Korea (2011-2016)
Source: PLoS One. 2020 Mar 4;15(3):e0229939. doi: 10.1371/journal.pone.0229939 (PMC7055891; doi:10.1371/journal.pone.0229939)
Supplement: S2 Table — (DOCX) [file pone.0229939.s002.docx]

**S2 Table. Univariate analysis of glacial acetic acid and age for mortality in intentional chemical ingestion**

|  | OR | 95% C.I. for EXP(B) | | Sig. |
| --- | --- | --- | --- | --- |
|  |  | Lower | Upper |  |
| Non-elderly, glacial acetic acid (-) | Reference |  |  | (-) |
| Non-elderly, glacial acetic acid (+) | 25.477 | 12.335 | 52.625 | <0.0001 |
| Elderly, glacial acetic acid (-) | 14.492 | 8.273 | 25.384 | <0.0001 |
| Elderly, glacial acetic acid (+) | 80.605 | 42.534 | 152.752 | <0.0001 |
